# Seroprevalence and clinical characteristics of viral hepatitis in transfusion-dependent thalassemia and hemophilia patients

**DOI:** 10.1371/journal.pone.0178883

**Published:** 2017-06-09

**Authors:** Tyng-Yuan Jang, Pei-Chin Lin, Ching-I Huang, Yu-Mei Liao, Ming-Lun Yeh, Yu-Sheng Zeng, Po-Cheng Liang, Wan-Yi Hsu, Shih-Pien Tsai, Zu-Yau Lin, Shinn-Cherng Chen, Jee-Fu Huang, Chia-Yen Dai, Chung-Feng Huang, Shyh-Shin Chiou, Wan-Long Chuang, Ming-Lung Yu

**Affiliations:** 1 Hepatobiliary Division, Department of Internal Medicine, Kaohsiung Medical University Hospital, Kaohsiung Medical University, Kaohsiung, Taiwan; 2 Division of Hematology and Oncology, Department of Pediatrics, Kaohsiung Medical University Hospital, Kaohsiung, Taiwan; 3 Special Hematologic Disease Service Center, Kaohsiung Medical University Hospital, Kaohsiung, Taiwan; 4 Faculty of Internal Medicine, School of Medicine, College of Medicine, Kaohsiung Medical University, Kaohsiung, Taiwan; 5 Department of Internal Medicine, Kaohsiung Municipal Hsiao-Kang Hospital, Kaohsiung Medical University, Kaohsiung, Taiwan; 6 Department of Nursing, Kaohsiung Medical University Hospital, Kaohsiung, Taiwan; 7 Department of Occupational Medicine, Kaohsiung Medical University Hospital, Kaohsiung Medical University, Kaohsiung, Taiwan; 8 Department of Preventive Medicine, Kaohsiung Medical University Hospital, Kaohsiung Medical University, Kaohsiung, Taiwan; 9 Institute of Biomedical Sciences, National Sun Yat-Sen University, Kaohsiung, Taiwan; 10 Liver Center, Division of Gastroenterology, Massachusetts General Hospital, Harvard Medical School, Boston, Massachusetts, United States of America; Centers for Disease Control and Prevention, UNITED STATES

## Abstract

**Background/Aims:**

Transfusion dependent subjects are at a great risk of viral hepatitis infection. We aimed to evaluate the prevalence and factors associated with hepatitis B virus (HBV) and hepatitis C virus (HCV) infection among transfusion-dependent patients in Taiwan.

**Methods:**

A total of 140 patients (67 thalassemic patients, 70 hemophilic patients, two patients with hereditary spherocytosis and one patient with von Willebrand disease) were prospectively enrolled to evaluate the prevalence and factors associated with viral hepatitis and spontaneous HCV clearance. All patients were tested for HBV and HCV serology and virology. Two consecutive serum samples, at least 1 year apart, were collected to clarify HCV seroclearance.

**Results:**

The seropositivity rate of hepatitis B surface antigen (HBsAg), HCV antibody (anti-HCV), and both HBsAg/anti-HCV were 6.4%, 45.7% and 5%, respectively. Logistic regression analysis of factors associated with anti-HCV seropositivity included age (odds ratio/95% confidence interval [OR/CI]: 1.12/1.07–1.18, P<0.001), serum alanine aminotransferase (ALT) (OR/CI: 1.04/1.02–1.06, P<0.001) and platelet counts (OR/CI: 0.995/0.991–0.998, P = 0.002). Age was the only factor independently associated with HBsAg seropositivity (OR/CI: 1.08/1.02–1.14.4, P = 0.007). Compared to patients born before 1992, the seroprevalence of HCV among thalassemic patients decreased dramatically in those born after 1992 (46.0% vs. 11.8%, p = 0.012). The seroprevalence of HCV among hemophilic patients also decreased significantly when comparing patients born before 1987 to those born after 1987 (79.5% vs. 11.5%, p<0.001). Similarly, the seroprevalence of HBV decreased significantly in the post-vaccination cohort compared to its counterpart (13.1%, vs. 1.3%, p = 0.005). The spontaneous clearance of HCV was observed in 25.4% (15/59) of patients, and ALT was the only factor associated with it (OR/CI 0.98/0.96–1.00, P = 0.02).

**Conclusions:**

Both HBV and HCV infections are prevalent among transfusion-dependent thalassemic and hemophilic patients in Taiwan. Nevertheless, seroprevalence decreased significantly and dramatically for HCV after universal blood screening and for HBV after implementation of a universal mass vaccination program.

## Introduction

Hepatitis B virus (HBV) and hepatitis C virus (HCV) infection are the major etiologies of chronic liver disease worldwide. More than 240 million people are chronically infected with HBV [1]. An estimated >185 million people globally are anti-HCV seropositive [2]. Both HBV and HCV have bloodborne transmission. Transfusion-dependent subjects, such as patients with thalassemia and hemophilia, are at a great risk of viral acquisition [3–7]. Post-transfusion HBV and HCV infection has resulted in a heavy liver disease burden in the past [8,9]. Given the viral inactivation of blood products [10] and anti-HCV screening of donated blood [9], HCV infection has decreased significantly in the last two decades [11]. Similarly, the prevalence of HBV infection has also dramatically decreased in the post-vaccination cohort. The risk of transfusion-transmitted viral hepatitis has been suggested to be less than 2.5 per 1 million donations in Western countries [12–14]. However, recent reports regarding the seroprevalence of viral hepatitis of the risk population in endemic countries such as Taiwan are scarce.

An estimated 25 to 52% of acute HCV infection are self-limiting [15]. A spontaneous HCV clearance rate of 28–42% has been reported among thalassemic and hemophilic patients in the West [16,17]. Host interleukin 28B (IL-28B) genetic variants [18,19] and concurrent HBV infection [19,20] have been considered as two of the important host and virological factors that determine spontaneous HCV clearance. The rate of clearance in the susceptible population in HBV endemic areas remains elusive. Taken collectively, the current study aimed to address the updated status of viral hepatitis B and C in the high-risk population in Taiwan. In addition, the rate of and factors associated with spontaneous HCV clearance among patients who have a higher proportion of favorable IL-28B genetic variants were also elucidated.

## Methods

### Patient enrollment

Transfusion-dependent patients were consecutively recruited in a Medical Center in Taiwan from May 2014 to November 2015. Patients received persistent blood product transfusions and regular follow-up laboratory testing at the interval of every two to eight weeks. Patients were tested for biochemistry, complete blood counts, HBV and HCV serology and virology, and host IL-28B genetic variants. Human immunodeficiency virus (HIV) test was mandatory and was determined by enzyme-linked immunosorbent assay (ELISA) (Architect HIV Ag/Ab Combo; Abbott Laboratories). HCV anti-bodies (Anti-HCV) were tested by a third-generation enzyme immunoassay (Abbott Laboratories, North Chicago, IL). HCV viral loads and genotype were determined by a real-time polymerase chain reaction assay (RealTime HCV; Abbott Molecular, Des Plaines IL, USA; detection limit: 12 IU/ml) [21]. Two consecutive serum samples, at least 1 year apart, were collected to confirm spontaneous HCV seroclearance by repeat HCV RNA testing in patients with anti-HCV seropisitivity. The HCV virology and liver biochemistry was presented as the time of study enrollment or before antiviral therapy if there is any. HBsAg was determined using a standard quantitative chemiluminescent microparticle immunoassay (ARCHITECT HBsAg, Abbott Diagnostics). Serum HBV DNA was tested using a standardized automated quantitative PCR assay (COBAS TaqMan HBV test, Roche Diagnostics, Branchburg, NJ; detection limit 12 IU/ml) [22]. The study was performed according to the guidelines of the International Conference on Harmonization for Good Clinical Practice. The ethics committee of the Kaohsiung Medical University Hospital has approved the protocol. All the patients provided informed consent before enrollment.

### IL28B genotyping

The IL28B rs8099917 genotype was selected as a candidate single nucleotide polymorphism (SNP) for the current study based on our previous studies [23–25]. Genotypes of the patients were determined using pre-designed ABI TaqManSNP genotyping assays (ABI Assay ID: C_11710096_10, Applied Biosystems, Foster City, CA, USA). PCR primers and two allele-specific probes were designed to detect the specific SNP target. The PCR reactions were performed in 96-well microplates with ABI 7500 real-time PCR (Applied Biosystems, Foster City, USA). Allele discrimination was achieved by detecting fluorescence using the System SDS software version 1.2.3.

### Statistical analyses

Frequency was compared between groups using the χ^2^ test with the Yates correction or the Fisher’s exact test. Group means were presented as the mean (standard deviation) and were compared using analysis of variance and Student’s *t*-testor the nonparametric Mann-Whitney test when appropriate. Stepwise logistic regression analysis was applied to assess the factors associated with anti-HCV seropositivity, spontaneous HCV clearance and HBsAg seropositivity by analyzing co-variant with P value < 0.1 in the univariate analysis. The statistical analyses were performed using the SPSS 20 statistical package (SPSS, Chicago, IL, USA). All statistical analyses were based on two-sided hypothesis tests with a significance level of *p*<0.05.

## Results

### Patient characteristics

A total of 140 patients were enrolled in the study. The mean age at the time of the study was 29 years (range: 12–62 of years), and men accounted for 74.3% (n = 104) of the population. The disease categories were mainly thalassemia (n = 67, 47.9%) and hemophilia (n = 70, 50.0%); 2 patients (1.4%) had hereditary spherocytosis and 1 patient (0.71%) had von Willebrand disease. Nine patients (6.4%) were seropositive for HBsAg (eight were hepatitis B e antigen [HBeAg] seronegative and the other patient’s HBeAg status was unavailable), whereas 64 patients (45.7%) were anti-HCV seropositive. Seven patients (5.0%) were seropositive for both HBsAg and anti-HCV. None of the patients had HIV infection. Ninety-two patients had an IL-28B genotype available, and theIL-28B rs8099917 TT genotype accounted for 91.1% of the patients (Table 1).

**Table 1:** Characteristics of the 140 patients and comparison of the clinical characteristics between subjects with thalassemia or hemophilia.

	All patients (n = 140)**	Thalassemia (n = 67, 47.9%)	Hemophilia (n = 70, 50%)	P value
Age (years, mean(SD))	29.0 (10.7)	26.2 (6.7)	32.2 (12.9)	0.001
Male, n (%)	104 (74.3)	33 (49.3)	70 (100)	<0.001
Body weight (kg, mean (SD))	56.7 (12.4)	52.4 (10.0)	67.8 (11.4)	<0.001
Anti-HCV (+), n (%)	64 (45.7)	25 (37.3)	38 (54.3)	0.13
HBsAg (+), n (%)	9 (6.4)	3 (4.5)	6 (8.6)	0.49
HBV DNA (log IU/mL, mean (SD))	2.96 (1.54)	3.04 (1.78)	2.92 (1.66)	0.94
AST (IU/L, mean (SD))	36.4(28.8)	42.7 (35.7)	31.2 (19.9)	0.02
ALT (IU/L, mean (SD))	49.2(60.5)	61.3 (79.1)	38.6 (33.2)	0.03
Serum creatinine ((mg/dL, mean (SD))	0.71(0.23)	0.60 (0.19)	0.83 (0.22)	<0.001
Hemogloblin (g/dL, mean (SD))	12.1 (2.8)	9.6 (1.3)	14.6 (1.4)	<0.001
Platelet count (x10^3^*u*/L, mean (SD))	307(150)	372 (178)	240 (72)	<0.001
Ferritin (ng/mL, mean(SD)), n = 114	1083.8 (2425.2)	1937.3 (3160.3)	150.6 (119.9)	<0.001
Diabetes, n/N (%)	9/104 (8.7)	8/47 (17.0)	1/54 (1.9)	0.02
HCV genotype, n/N (%)				
1	30/44 (68.2)	18/21 (85.7)	12/23(52.2)	0.02
Non-1	14/44 (31.8)	3/21 (14.3)	11/23(47.8)	
IL-28B rs8099917 TT genotype, n/N(%)	92/101(91.1)	43/48 (89.6)	46/50 (92)	0.79
HCV RNA (log IU/mL, mean (SD))	5.25 (1.18)	4.87 (1.15)	5.60 (1.12)	0.04

SD: standard deviation; AST: aspartate aminotransferase; ALT: alanine aminotransferase. HBsAg: Hepatitis B surface antigen. Anti-HCV: hepatitis C antibody. IL-28B: Interleukin28B.

** The patients other than hemophilia and thalassemia were with hereditary spherocytosis (n = 2) and von Willebrand disease (n = 1)

### Differences in the clinical characteristics between subjects with thalassemia and hemophilia

Compared to hemophilia patients, thalassemia patients were younger, had higher levels of serum AST, ALT, ferritin and platelet counts and lower levels of serum hemoglobin and HCV RNA. Thalassemia patients also had higher proportions of HCV-1 and diabetes mellitus. The seroprevalence of viral hepatitis did not differ between the two patient groups (Table 1).

### Factors associated with anti-HCV and HBsAg seropositivity

**Fig 1:**
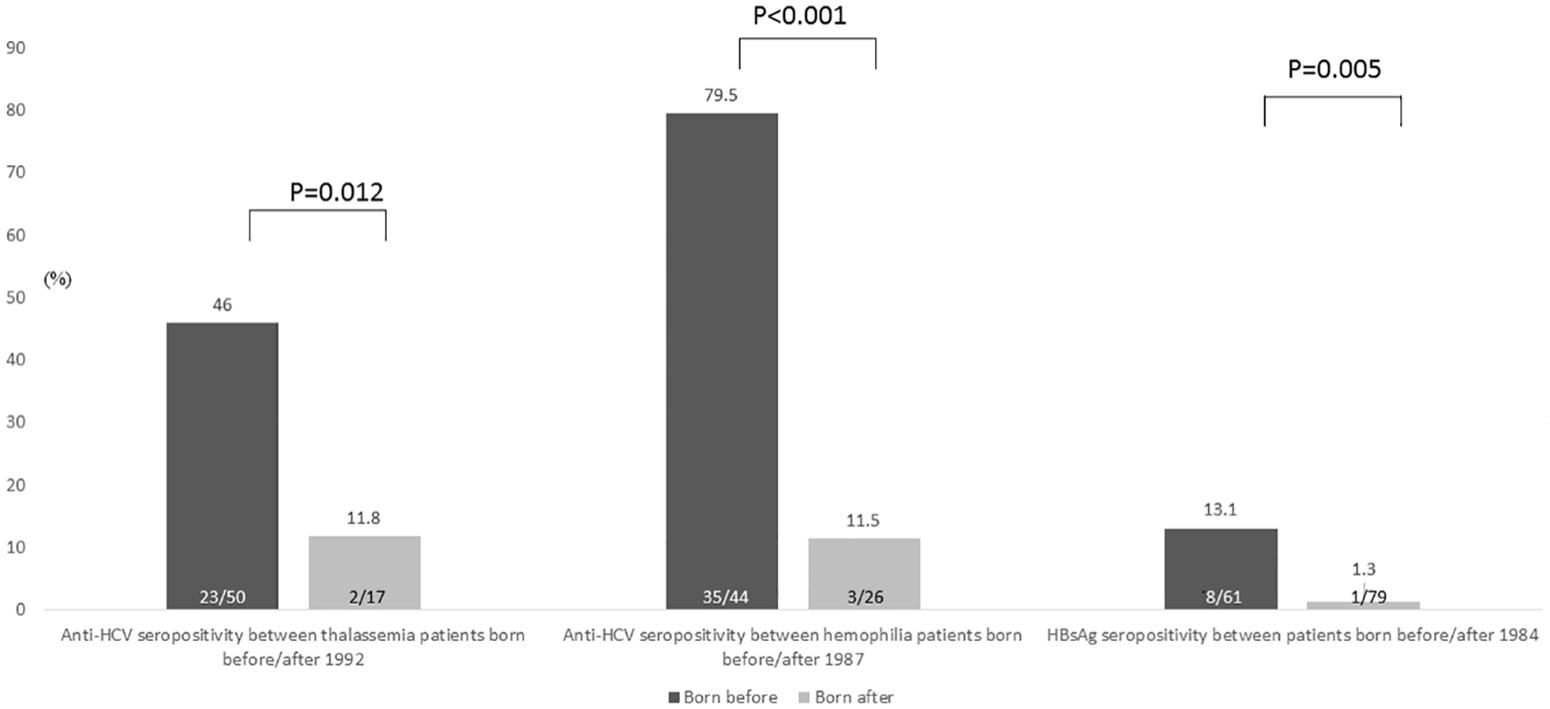
Seroprevalence of anti-HCV and HBsAg seropositivity in thalassemic and hemophilic patients with different age groups.

Univariate analysis revealed that factors associated with anti-HCV seropositivity included older age, higher levels of serum aspartate aminotransferase (AST) and alanine aminotransferase (ALT) and lower platelet (PLT) counts. Thalassemic patients who born before 1992 had a higher rate of anti-HCV seropositivity than those who born after 1992 (46.0% vs. 11.8%, P = 0.012). Similarly, hemophilic patients born before 1987 had a higher rate of anti-HCV seropositivity than those born after 1987 (79.5% vs. 11.5%, P<0.001) (Fig 1). Multivariate analysis of factors associated with anti-HCV seropositivity included age (odds ratio [OR]/95% confidence intervals [CI]: 1.12/1.07–1.18, P<0.001), ALT level (OR/CI: 1.04/1.02–1.06, P<0.001) and PLT counts (OR/CI: 0.995/0.991–0.998, P = 0.002) (Table 2). Univariate analysis of factors associated with HBsAg seropositivity included older age, specifically subjects who were born before 1984. Patients born before 1984 had a higher rate of HBsAg seropositivity than those who born after 1984 (13.1% vs. 1.3%, P = 0.005) (Fig 1). Multivariate analysis demonstrated that age was the only factor associated with HBsAg seropositivity (OR/CI: 1.08/1.02–1.14.4, P = 0.007) (Table 3).

**Table 2:** Univariate and multivariate analysis of factors associated with anti-HCV seropositivity.

	Anti-HCV(+) (n = 64, 45.7%)	Anti-HCV(-) (n = 76, 54.3%)	P value	Logistic regression analysis		
				OR	95% CI	*P* value
Age (years, mean(SD))	34.0 (10.3)	24.4 (8.9)	<0.001	1.12	1.07–1.18	<0.001
Born before 1992, n (%)	60 (93.8)	43 (56.6)	<0.001			
Male gender, n (%)	49 (47.1)	55 (52.9)	0.57			
Body weight (kg, mean (SD))	57.5 (11.8)	56.1 (13.0)	0.62			
Thalassemia/hemophilia, n (%)	25/38 (39.1/60.9)	42/32 (55.3/44.7)	0.13			
AST (IU/L, mean (SD))	49.6 (36.1)	24.9 (11.8)	<0.001			
ALT (IU/L, mean (SD))	77.2 (79.5)	25.7 (15.9)	<0.001	1.04	1.02–1.06	<0.001
Serum creatinine ((mg/dL, mean (SD))	0.72 (0.23)	0.71 (0.22)	0.81			
Hemoglobin (g/dL, mean (SD))	12.7 (2.7)	11.6 (2.9)	0.02			
Platelet count (x10^3^*u*/L, mean (SD))	270 (153)	339 (140)	0.007	0.995	0.991–0.998	0.002
HBsAg (+), n (%)	7 (10.9)	2 (2.6)	0.08			
HBV DNA (log IU/mL, mean (SD))	2.77 (1.73)	3.42 (1.24)	0.66			
IL-28B rs8099917 TT genotype, n/N(%)	48/54 (88.9)	44/47 (93.6)	0.41			

SD: standard deviation; OR: odds ratio; CI: confidence intervals; AST: aspartate aminotransferase; ALT: alanine aminotransferase. HBsAg: Hepatitis B surface antigen. Anti-HCV: hepatitis C antibody. IL-28B: Interleukin 28B.

**Table 3:** Differences of characteristics between subjects with or without HBsAg seropositivity.

	HBsAg (+) (n = 9, 6.4%)	HBsAg (-) (n = 131, 93.6%)	P value	Logistic regression analysis		
				OR	95% CI	*P* value
Age (years, mean(SD))	40.0 (10.7)	28.4 (10.4)	0.02	1.08	1.02–1.14	0.007
Male gender, n (%)	7 (87.5)	97 (73.5)	0.38			
Born before 1984, n (%)	8 (88.9)	53 (40.5)	0.01			
Body weight (kg, mean (SD))	62.7 (26.6)	56.5 (11.8)	0.40			
Thalassemia/hemophilia, n (%)	2/6 (22.2/66.7)	65/64 (49.6/48.9)	0.34			
AST (IU/L, mean (SD))	49.1(35.6)	35.5(28.2)	0.17			
ALT (IU/L, mean (SD))	61.1 (67.1)	48.4 (60.3)	0.59			
Serum creatinine ((mg/dL, mean (SD))	0.67 (0.22)	0.71 (0.23)	0.64			
Hemoglobin (g/dL, mean (SD))	12.8 (3.0)	12.1 (2.8)	0.49			
Platelet count (x10^3^*u*/L, mean (SD))	288 (21)	309 (146)	0.71			
IL-28B rs8099917 TT genotype, n/N(%)	4/4 (100)	88/97 (90.7)	0.52			
Anti-HCV(+)	7 (77.8)	57 (43.5)	0.08			
HCV RNA (log IU/mL, mean (SD))	5.07 (1.27)	5.27(1.19)	0.82			

SD: standard deviation;OR: odds ratio; CI: confidence intervals; AST: aspartate aminotransferase; ALT: alanine aminotransferase.HBsAg: Hepatitis B surface antigen. Anti-HCV: hepatitis C antibody. IL-28B: Interleukin-28B.

### Rate and factors associated with spontaneous HCV clearance

**Fig 2:**
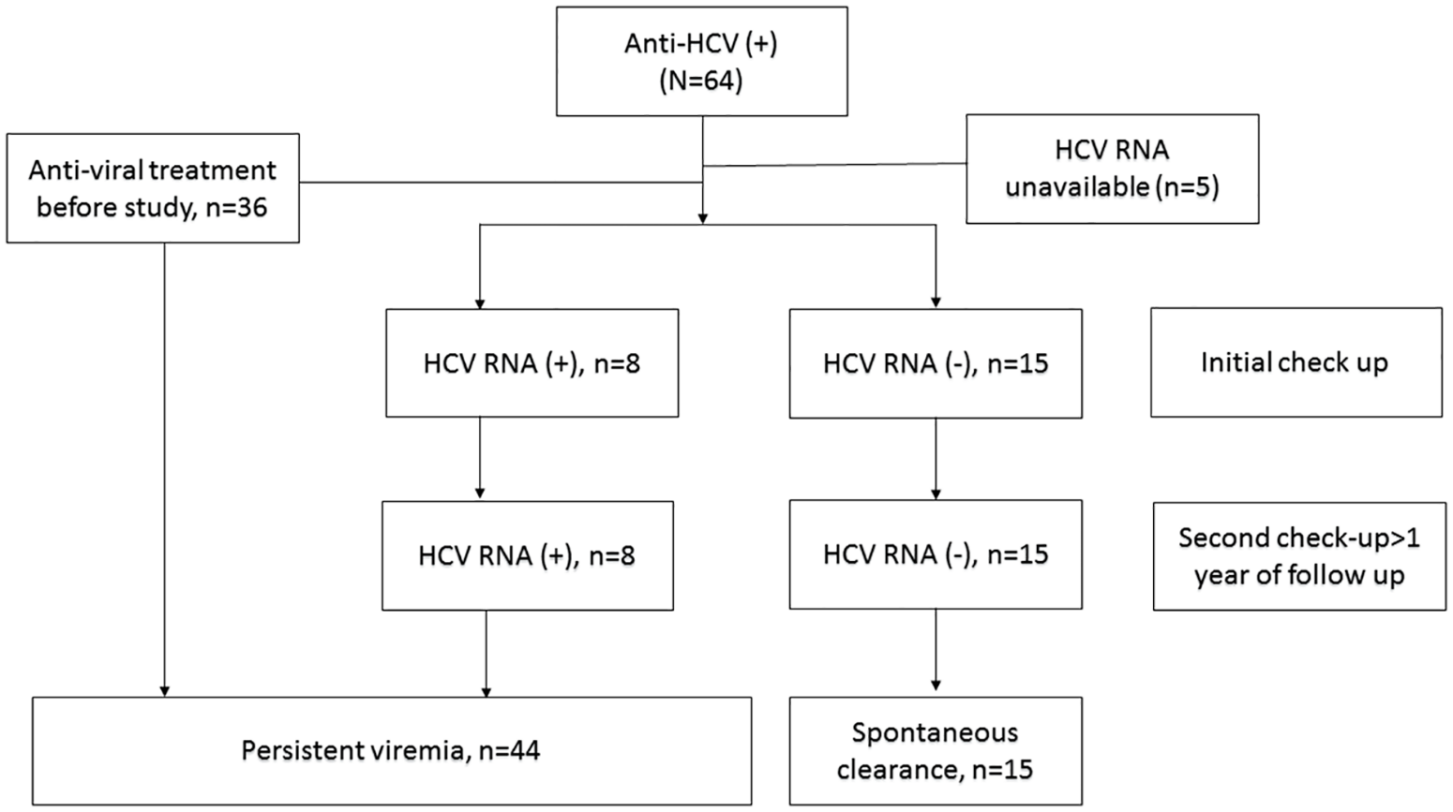
Flow chart of the patients with anti-HCV seropositivity.

We further explored the rate and factors associated with spontaneous HCV clearance in patients with anti-HCV seropositivity. As shown in Fig 2, thirty-six patients who had the history of antiviral therapy before study were regarded as those with persistent viremia. After excluding 5 patients whose HCV RNA were not available, eight patients had detectable HCV RNA whereas the other 15 were with undetectable HCV RNA at the time of enrollment. Taken collectively, Forty-four of the 59 patients had persistent HCV viremia (74.6%), and the spontaneous clearance rate was 25.4% (n = 15). Among the HCV viremic patients, the mean HCV RNA level was 5.25 log IU/mL, and HCV genotypes 1, 2 and 6 accounted for 68.2%, 29.5%, and 2.3% of cases, respectively. Univariate analysis revealed that patients with spontaneous HCV clearance had lower levels of AST and ALT. Multivariate analysis demonstrated that ALT was the only factor associated with HCV spontaneous seroclearance (OR/CI 0.98/0.96–1.00, P = 0.02). (Table 4).

**Table 4:** Factors associated with spontaneous HCV seroclearance in 59 anti-HCV (+) patients.

	Spontaneous HCV seroclearance (n = 15, 25.4%)	HCV viremia (n = 44, 74.6%)	P value	Logistic regression analysis		
				OR	95% CI	*P* value
Age (years, mean(SD))	34.8 (10.2)	33.3 (9.9)	0.62			
Male gender, n (%)	11(73.3)	33 (75)	0.90			
Thalassemia/hemophilia, n (%)	4/10(26.7/66.7)	21/23 (47.7/52.3)	0.10			
Born before 1992, n (%)	13 (86.7)	42 (95.5)	0.24			
AST (IU/L, mean (SD))	35.2 (34.0)	55.8 (36.3)	0.06			
ALT (IU/L, mean (SD))	39.0 (42.4)	95.4 (89.8)	0.002	0.98	0.96–1.00	0.02
Serum creatinine ((mg/dL, mean (SD))	0.72 (0.21)	0.68 (0.18)	0.47			
Hemoglobin (g/dL, mean (SD))	13.0 (2.9)	12.7 (2.6)	0.70			
Platelet count (x10^3^*u*/L, mean (SD))	301(132)	269 (164)	0.44			
HBsAg (+), n (%)	3 (20.0)	3 (6.8)	0.15			
HBV DNA (log IU/mL, mean (SD))	3.41 (2.15)	1.68 (1.45)	0.30			
HBV DNA detectatble,** n (%)	3 (20.0)	2 (4.5)	0.06			
IL-28B rs8099917 TT genotype, n/N (%)	12/12 (100)	36/42 (85.7)	0.17			

SD: standard deviation; OR: odds ratio; CI: confidence intervals; BMI: body mass index; AST: aspartate aminotransferase; ALT: alanine aminotransferase. HBsAg: Hepatitis B surface antigen. Anti-HCV: hepatitis C antibody. IL-28B: Interleukin 28B * history and data available in 51 patients.

**Detectatble HBV DNA defined as >20 IU/mL

## Discussion

We demonstrated that anti-HCV seropositivity in transfusion-dependent patients currently remains high in Taiwan. More than one-third of thalassemic patients and half of hemophiliac patients were infected with HCV. However, the rate of HCV infection decreased dramatically after universal blood screening. One-fourth of the anti-HCV seropositive patients had a self-limiting infection after acute HCV infection. On the other hand, the prevalence of HBV infection was similar to the general population in Taiwan and was very rare in the post-vaccination cohort.

A universal vaccination program for HBV for infants was launched in Taiwan in 1984. The carrier rate of HBV in children covered by the program has decreased from 15% to <1% since then [26–28]. We have previously reported a higher HBV infection rate in post-vaccination indigenous residencies [29]. However, we noted only 1.3% of the population had HBV infection in the post vaccination cohort. The fact once again indicates the success of the universal HBV vaccination program in Taiwan. The result also highlighted the minimal risk of HBV transmission through horizontal pathways among members of the high-risk group.

The prevalence of anti-HCV seropositivity has geographical variations ranging from 0.02% to 22% worldwide [15]. Taiwan is an endemic country with a seroprevalence ranging from 3.3% to 4.4% [30–34]. Of particular note is that the prevalence of HCV infection prevails in certain areas [32] and populations [19,35,36]. The prevalence of anti-HCV seropositivity among thalassemia patients has been reported from 2.7% to 97% across countries in an early report [37]. Routine anti-HCV screening of donated blood started in July 1992 in Taiwan. It has been suggested that 43% of poly-transfused thalassemic children were infected with HCV near the time of initiating blood screening in Taiwan [9]. The prevalence was similar to our finding that approximately half of the thalassemia patients born before 1992 were anti-HCV seropositive.

For the hemophilia patients, viral inactivated factor concentrates were not available until 1985, and recombinant products became available in 1989 [10,38]. Most hemophiliac patients treated with plasma products before 1987 had a high risk of HCV infection [39], and the prevalence ranged from 62% to 90% in the 1990s [40]. It has been suggested that up to 90% of hemophilia patients were infected with HCV in Taiwan before 1991 [41], which was similar to our finding that 80% of hemophilic patients born before 1987 were infected with HCV. Reports regarding the recent prevalence of HCV infection among transfusion-dependent subjects are rare in Taiwan. We observed a dramatically decreased HCV seroprevalence in the post-screening cohort. However, one-tenth of the at-risk population, either hemophilia or thalassemia patients, remains infected with HCV. It is unclear whether the patients acquired HCV through the window-period of blood transfusion or through other transmission routes. With the improvement of public health education [32] and the initiation of nucleic acid amplification testing (NAT) [42] in Taiwan, it is anticipated that the seroprevalence of HCV infection among the population will further decrease in the future.

The demands of blood products transfusion vary, which depended on individual disease characteristics and disease severities [43,44]. In general, thalassemic patients receive packed red blood cell transfusion at the interval of every 2–4 weeks. Hemophilic patients received clotting factor prophylaxis with the frequency of once to twice weekly. Unlike thalassemic patients who receive single donor of packed RBC, the clotting factor per unit may derive from hundreds of subjects. This may be in part responsible for a ultimately higher HCV infection rate among hemophilic than thalassemic patients in pre-screen era.

The rate of spontaneous seroclearance of HCV varied from 10–52% in the general population. A similar rate of 28–42% has been reported in hemophilia and thalassemia patients [16,17]. We noted a spontaneous clearance rate of 25.4% in this cohort, which is not far from that of the general population [45]and uremic patients in Taiwan [19]. Several factors have been associated with self-limited HCV infection. Among them, IL-28B genetic polymorphisms have been viewed as the strongest host factor [18,46,47]. We have also shown that concurrent HBV infection may enhance HCV clearance, possibly due to reciprocal viral interference [19]. In the current study, we failed to prove that favorable IL-28B genotype carriage and concurrent HBV infection had higher rate of HCV seroclearance. The sample size may be too small for the statistics to be significant. Further studies enrolling more Taiwanese patients may be warranted to clarify this issue.

Patients were recruited in one of the largest medical centers in Taiwan, which is responsible for the major task of transfusion clinical service. In addition, it is noteworthy that all patients were prospectively recruited and constantly followed. Owing to the unique characteristics of the population, selection bias may exist but be limited. The current study also failed to quantify the number of exposures in terms of spontaneous clearance. Age and etiology (hemophilia versus. thalassemia) were the most important factors associated with potential viral exposures. However, we did not observed their associations with spontaneous clearance in patients with anti-HCV seropositivity. In conclusion, the prevalence rates of HBV and HCV infections among transfusion-dependent patients were very high in Taiwan in the past. However, the seroprevalence decreased significantly and dramatically in the HCV-post screening and HBV-post vaccination era. Further large-scale nationwide surveillance may be warranted to validate the current study.
